# High Levels of Soluble Ctla-4 Are Present in Anti-Mitochondrial Antibody Positive, but Not in Antibody Negative Patients with Primary Biliary Cirrhosis

**DOI:** 10.1371/journal.pone.0112509

**Published:** 2014-11-10

**Authors:** Daniele Saverino, Giampaola Pesce, Princey Antola, Brunetta Porcelli, Ignazio Brusca, Danilo Villalta, Marilina Tampoia, Renato Tozzoli, Elio Tonutti, Maria Grazia Alessio, Marcello Bagnasco, Nicola Bizzaro

**Affiliations:** 1 Department of Experimental Medicine – Section of Human Anatomy, University of Genova, Genova, Italy; 2 Autoimmunity Unit, Department of Internal Medicine, University of Genova, Genova, Italy; 3 Department of Internal Medicine, University of Siena, Siena, Italy; 4 Department of Clinical Pathology, Buccheri La Ferla Hospital, Palermo, Italy; 5 Allergology and Clinical Immunology, S. Maria degli Angeli Hospital, Pordenone, Italy; 6 Laboratory of Clinical Pathology, University Hospital, Bari, Italy; 7 Clinical Pathology, Department of Laboratory Medicine, S. Maria degli Angeli Hospital, Pordenone, Italy; 8 Immunopathology and Allergology Unit, S. Maria della Misericordia Hospital, Udine, Italy; 9 Department of Laboratory Medicine, Biochemistry Laboratory, Riuniti Hospital, Bergamo, Italy; 10 Laboratory of Clinical Pathology, San Antonio Hospital, Tolmezzo, Udine, Italy; Emory University School of Medicine, United States of America

## Abstract

Primary biliary cirrhosis (PBC) is a chronic autoimmune cholestatic liver disease frequently characterized by anti-mitochondrial autoantibodies (AMA). A minority of patients are AMA-negative. Cytotoxic-T-Lymphocyte-Antigen-4 (CTLA-4) is a surface molecule expressed on activated T-cells delivering a critical negative immunoregulatory signal. A soluble form of CTLA-4 (sCTLA-4) has been detected at high concentrations in several autoimmune diseases, and its possible functional meaning has been suggested. We aimed to evaluate sCTLA-4 concentration in sera of patients with PBC and to correlate it to immunological abnormalities associated with the disease. Blood samples were collected from 82 PBC-patients diagnosed according to international criteria (44 AMA-positive/MIT3-positive and 38 AMA-negative-MIT3-negative), and 65 controls. sCTLA-4 levels were evaluated by ELISA and Western blot. Increased sCTLA-4 concentrations were found in all AMA-positive PBC-patients, but in none of the AMA-negative ones, nor in normal controls or in controls with unrelated liver diseases. sCTLA-4 presence was associated with autoantibodies against MIT3, but not with nuclear autoantibodies (sp100, gp210). This is the first study to demonstrate that levels of sCTLA-4 are elevated in sera of PBC patients. However, they are clearly restricted to patients with AMA positivity, suggesting an immunological difference with respect to AMA-negative ones.

## Introduction

Primary biliary cirrhosis (PBC) is a chronic cholestatic liver autoimmune disease characterized by slow progressive immune-mediated destruction of the small- and medium-sized bile ducts, leading to liver fibrosis, cirrhosis, eventually requiring transplantation [1]–[6]. The prevalence of PBC ranges from 30 to 400 per million. PBC preferentially affects middle-aged women (m:f ratio 9 to 1), sharing this characteristic with other autoimmune diseases [7]. Progression of PBC is usually slow-paced, but symptoms of portal hypertension and hepatic decompensation (jaundice, ascites, or variceal bleeding) may develop several years after the initial diagnosis [1], [2], [4], [8], [9]. The aetiology of PBC is unknown; however, it is believed that genetic susceptibility, and environmental factors are involved in concert [10]. Studies in animal models suggest that specific infectious and environmental triggers can induce PBC-specific pathological features, probably by the mechanism of molecular mimicry [10]–[14]. This would occur in the setting of T regulatory impairment, particularly in susceptible individuals [15], [16].

The autoimmune pathogenesis of PBC is supported by a large amount of experimental and clinical data, such as the presence of autoreactive T cells, and serum autoantibodies characteristic of the disease [17]–[19]. High-titer serum AMA positivity is pathognomonic for PBC, and is present in 90–95% of patients [8], [17]–[19]. Patients lacking detectable AMA but presenting signs and symptoms of PBC should be considered as having “AMA-negative PBC”. AMA are specific to the lipolylated domains within components of the 2-oxoacid dehydrogenase family of enzymes, particularly the E2 component of the pyruvate dehydrogenase complex (PDC-E2) [17]–[19]. In addition to AMA, PBC-specific anti-nuclear autoantibodies (ANA) including the “multiple nuclear dot” and “nuclear membrane/rim” patterns are present in approximately 30% of patients [17]–[19]. The “multiple nuclear dot” pattern corresponds to autoantibodies against sp100, sp140, promyelocytic leukaemia nuclear body proteins, and small ubiquitin-like modifiers [20]. The “nuclear envelope/rim” pattern corresponds to reactivity specific for gp210 and nucleoporin p62 [21]. Up to 30% of PBC patients have both patterns, which demonstrate significant disease specificity. PBC-specific ANA may be present in AMA-negative PBC patients, in asymptomatic individuals and in family members of PBC patients [17]–[19].

Whether AMA-negative PBC and AMA-positive PBC are the same or two immunologically different diseases is debated. The use of more sensitive techniques other than immunofluorescence, such as ELISA and immunoblotting, has revealed that a number of patients who were classified as AMA-negative by immunofluorescence assay, display a positivity for AMA related autoantigens. This would suggest that the different expression of AMA refers to a single autoimmune disorder and that the AMA-negative PBC cohort results from analytical limits. Nevertheless, a small proportion of PBC patients that are definitely AMA-negative, whatever the detection method employed, does exist. [19].

In general, the activity of T cells is controlled by several activatory and inhibitory co-receptors. Among various inhibitory pathways, Cytotoxic-T-Lymphocyte-Antigen-4 (CTLA-4), a member of the immunoglobulin superfamily, plays a key role in restraining T-cell responses during antigenic stimulation. CTLA-4 is homologous to the activating coreceptor CD28, but its affinity for CD80 and CD86 molecules is much higher than that of CD28. Thus, it can efficiently compete with CD28 for its ligands and maintain homeostasis during T-cell responsiveness [22]–[24]. CTLA-4 has steady-state messenger RNA (mRNA) levels for two known isoforms: a full-length isoform (flCTLA-4) encoded by exon 1 (leader peptide), exon 2 (ligand-binding domain), exon 3 (transmembrane domain) and exon 4 (cytoplasmic tail) and a soluble form (sCTLA-4), which lacks exon 3. sCTLA-4, originating from alternative splicing, results in the loss of a cysteine residue and is found in the serum as a soluble monomeric protein [24]–[26]. The presence of high concentrations of sCTLA-4 was observed in sera of patients with autoimmune thyroid diseases [26], [27], as well as in patients with other autoimmune diseases, such as type-1 diabetes, diffuse cutaneous systemic sclerosis [28], systemic lupus erythematosus [29] and myasthenia gravis [30], [31].

As for other diseases of autoimmune origin, polymorphisms of *CTLA-4* (particularly, CTLA-4+49G>A polymorphism) could play a role on susceptibility to PBC [32].

Although our understanding of the role of the *CTLA-4* gene and its protein products is incomplete, we analysed the presence of sCTLA-4 in sera from PBC patients, under the hypothesis that its levels could be related to the immunological abnormalities associated with the disease. Furthermore, we separately studied a consistent group of clinically and histologically diagnosed PBC patients selected on the basis of AMA negativity, and compared the results obtained with those of AMA-positive patients. The results showed relevant differences between the two groups.

## Materials and Methods

### Patients

Blood samples were collected from PBC patients from different regions of Italy, following the ethical guidelines of the most recent Declaration of Helsinki (Edinburg 2000) and all patients enrolled in this study provided written informed consent. The Ethics Committee of Azienda Ospedaliero-Universitaria di Udine, Italy, approved the research. Ninety % of them were female with a median age of 61 years (range 37–89).

The diagnosis of PBC was formulated by experienced gastroenterologists from 2006 to 2008. For each patient at least two out of three internationally accepted criteria were fulfilled, i.e., presence of serum AMA, increased enzymes indicating cholestasis (i.e., alkaline phosphatase) for longer than 6 months, and compatible or diagnostic liver histology [8], [17]–[19]. All AMA-negative patients (see below) fulfilled the latter two criteria. All sera were collected at diagnosis before starting any kind of specific medication.

The study population was composed by two distinct groups of PBC sera. The first included 44 AMA-positive PBC sera from consecutively diagnosed patients. As a second group, 46 sera were randomly selected from a previously described cohort of 100 AMA-negative PBC cases [19]. Among them, 8 were excluded on the basis of positivity for MIT3 (see below): thus, 38 “true” AMA-negative patients were eventually included.

An overlap syndrome with systemic sclerosis was observed in only 2 out of the total pool of PBC patients studied.

Sera from 20 patients with hepatitis B virus infection and 45 blood donor volunteers (17 and 36 women, aged 32–64 and 30–61 years, respectively) were included as controls. Sera were stored frozen until the use and freezing and thawing was avoided.

### Methods

Serum samples were first assayed for AMA and ANA by IIF on sections of rat kidney, stomach and liver, and on HEp2 cells, respectively (test kits from EUROIMMUN, Lübeck, Germany, and INOVA Diagnostics, San Diego, CA, USA) and by an ELISA screening test (PBC Screen, INOVA Diagnostics, and EUROIMMUN) using three coating antigens: recombinant pMIT3 and purified gp210 and sp100 with anti-IgG and anti-IgA dual conjugate. The manufacturer’s cut-off was established at 25 units. Samples were considered as negative (≤20.0 units), equivocal (20.1–24.9 units), or positive (≥25.0 units). A negative result indicated the absence of antibodies to MIT3, gp210, or sp100. A positive result indicated the presence of antibodies to one or more of the antigens included in the PBC Screen assay. Subsequently, sera from patients with PBC were tested on the individual MIT3, gp210 and sp100 IgG Quanta Lite ELISAs (INOVA Diagnostics) [19].

### sCTLA-4 measurement

A specific ELISA method was used for measuring serum sCTLA-4 levels (Bender Med System, Prodotti Gianni SpA, Milano, Italy), according to the manufacturer’s protocol. Each sample was diluted 1∶10 in assay buffer provided by the manufacturer and tested in triplicate. Deviation between triplicates was <10% for any reported value. The lowest sensitivity threshold was 0.1 ng/ml.

The analytical response was linear approximately between 0.162 and 1.200 of absorbance values (corresponding to 0.1–50 ng/ml) as assessed by serial dilution test using a strongly positive serum (data not shown). For samples with sCTLA-4 concentration higher than 50 ng/ml, the ELISA tests were repeated using a greater dilution factor (1∶100) [27].

Western blotting was used to confirm the presence of sCTLA-4 by testing four different samples (three positive and one negative by ELISA). Proteins were separated by loading 200 µl of sera on 10–20% gradient PAGE in a discontinuous buffer system on a Mini-Protean system (Bio-Rad, Segrate, Milano, Italy). The separated components were electroblotted onto polyvinylidene difluoride membranes. The blots were washed with 0.15 M NaCl, 0.05 M Tris (TBS), pH 7.5, with 0.3% Tween 20 and reacted with a 1∶100 dilution of the anti-CTLA-4 mAb (clone 14D3, IgG2a, eBiosciences, San Diego, CA) for 1 hour at room temperature, washed, and then reacted with reporter antibody (HRP-conjugated anti-mouse IgG) for 1 hour. The blots were then developed by the use of a commercially available chemiluminescence detection kit (BMB, Indianapolis, IN) according to the manufacturer’s instructions.

### Statistical analysis

Statistical analysis was performed using the Mann–Whitney U-test for comparison of sCTLA-4 levels among AMA-positive and AMA-negative patients. Spearman regression analysis was used to evaluate the correlation between sCTLA-4 and other parameters (such as PBC Screen, MIT3, gp210, sp100, alkaline phosphatase (ALP), gamma–glutamyl transpeptidase (gammaGT), alanine aminotransferase (ALT), total and direct bilirubin, albumine, and Scheuer stage). A *P* value less than 0.05 was considered statistically significant. All analyses were performed by using the GraphPad Prism4 software 4.0 (GraphPad Software Inc., La Jolla, CA).

## Results

### Serological characteristics of PBC patients

All IIF AMA-positive PBC sera (44/44) tested positive at the PBC Screen assay. When single antigens were used, all sera recognized MIT3, whereas none recognized gp210 alone, nor sp100 alone.

Among IIF AMA-negative PBC sera, 25/38 tested positive at the PBC Screen assay. When single antigens were used, none of 38 sera recognized MIT3 alone, 2/38 recognized gp210 only, and 8/38 sp100. Finally, no serum reacted against two different antigens.

As expected, the difference of PBC screen, gp210 and sp100 between the two groups of AMA-IIF positive and AMA IIF-negative PBC patients was statistically significant, p<0.05 (Table 1). In fact, our previous data confirm the hypothesis that a substantial part of IIF AMA-negative (a so called “probable”) PBC cases manifest disease-specific autoantibodies when tested using newly available tools [19].

**Table 1:** Biochemical, serological and histological features in AMA-positive and in AMA-negative PBC patients (values are expressed as median and range).

	AMA-positive (n = 44)	AMA-negative (n = 38)	P value
Women (*n*)	38	33	n.s.
Age (*years*)	38–82	37–82	n.s.
PBC screen (*units*)	152.44 (78–200)	39.46 (2–119)	<0.0001
gp210 (*units*)	4 (1–11)	2 (1–33)	<0.0001
sp100 (*units*)	4 (2–16)	3 (1–179)	0.0064
ALP (*UI/l*)	289 (147–2675)	302 (138–2943)	n.s.
gammaGT (*U/l*)	156 (37–1335)	140 (39–1822)	n.s.
ALT (*UI/l*)	61 (37–240)	40 (32–555)	n.s.
Total bilirubin (*mg/dl*)	1.7 (0.8–2.91)	0.8 (0.3–41.4)	n.s.
Direct bilirubin (*mg/dl*)	0.9 (0.3–1.6)	0.2 (0.1–4.7)	n.s.
Albumin (*g/l*)	4.4 (3–5.8)	4 (2.9–5.1)	0.0751
Histological Scheuer stage	III (I–IV)	II (I–IV)	n.s.
I	11 patients	10 patients	
II	6 patients	18 patients	
III	16 patients	1 patient	
IV	11 patients	4 patients	
sCTLA-4 (*ng/ml*)	14.3 (0.9–81.8)	1 (0.1–15.1)	<0.0001
	**Hepatitis B virus infection** (n = 20)	**Healthy donors** (n = 45)	**P value**
Women (*n*)	17	36	n.s.
Age (*years*)	32–64	30–61	n.s.

As shown in Table 1, increased enzyme values (i.e., ALP, ALT, and gammaGT) indicating cholestasis and stage determination did not reveal a statistical difference between the two groups of patients, though the majority of AMA-positive PBC patients were in stage 3 (characterized by fibrous scaring bridging portal tracts with occasional foci of bile duct loss), whereas AMA-negative were in stage 2 (showing portal enlargement with bile ductular reaction and inflammatory cell infiltration).

Finally, none of the control sera showed elevation of ALP or positivity for AMA.

### sCTLA-4 is present in sera from PBC patients and its level is related to AMA

**Figure 1:**
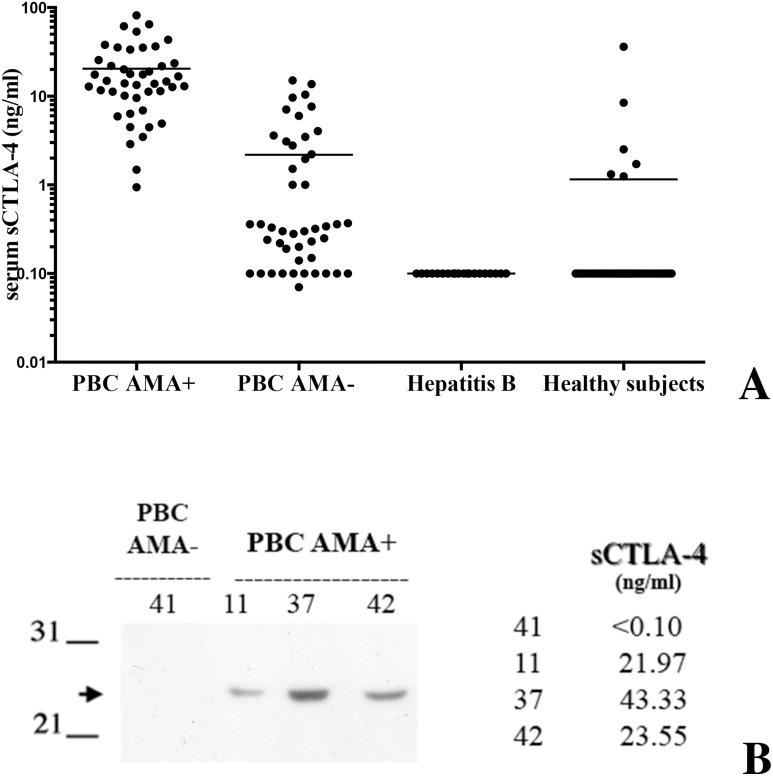
A sCTLA-4 is found in serum of PBC AMA-positive patients. Panel A, The concentration of sCTLA-4 was evaluated by ELISA on sera collected from AMA-positive PBC patients, AMA-negative PBC patients, and hepatitis B-infected patients and healthy donors as controls. Results are expressed as nanogram per milliliter. Each sample was diluted 1∶10 and tested in triplicate. Samples showing values higher then the detection limit of the test (50 ng/ml) were diluted appropriately and tested again. Deviation between triplicates was <10% for any reported value. Panel B, Immunoprecipitation of sCTLA-4 was performed on sera from a representative group of AMA-positive PBC patients. In addition, a serum undetectable sCTLA-4 dosing (below the sensibility of the text) was shown. Arrow marks 23 kDa species.

We assessed the presence of the soluble form of CTLA-4 in sera from PBC patients and controls by using a sensitive ELISA. sCTLA-4 levels were markedly raised in virtually all of AMA-positive PBC patients at diagnosis and significantly higher compared to AMA-negative PBC patients and controls (p<0.001) (Fig. 1A). Only 6 out 45 healthy subjects and none of the 20 hepatitis B virus-infected patients had detectable sCTLA-4. No correlation was found between sCTLA-4 levels and age or presence of symptoms.

We confirmed the presence of sCTLA-4 by Western-blot testing three different samples that were positive by ELISA. As shown in Fig. 1B, a characteristic band of approximately 23 kDa is apparent, which is consistent with previous reports [26], [27]. For comparison, a serum undetectable amount of sCTLA-4 is shown.

### Correlation among sCTLA-4 and classical PBC clinical and functional parameters

As observed above, the levels of sCTLA-4 strongly correlated with AMA-positivity. For this reason we compared serum sCTLA-4 levels to other specific autoantibodies characteristically present in PBC patients. Otherwise, all the other clinical/functional parameters analysed failed to show any significant correlation with serum sCTLA-4 levels. Similarly, no correlation was found between sCTLA-4 levels and sp100 or gp210 positivity**.** As far as sp100 is concerned, out of 8 sp100-positive sera, 4 had undetectable, and 4 had detectable sCTLA-4.

**Figure 2:**
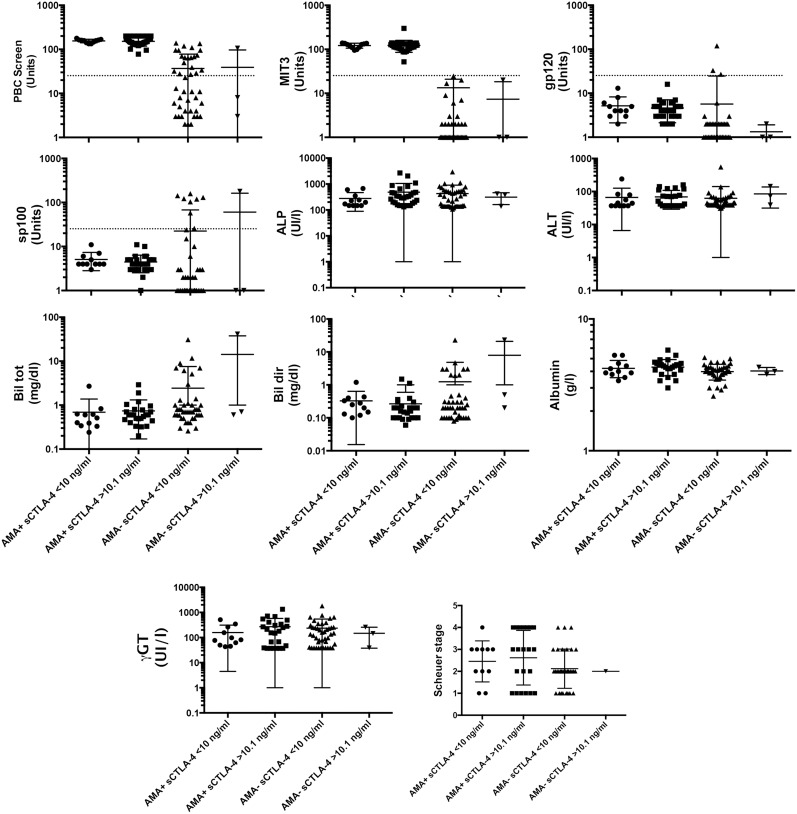
Correlation of sCTLA-4 amounts and cholestatic liver biochemistry, histology, and serum anti-mitochondrial autoantibodies. The AMA-positive and AMA-negative patients were divided in two subgroups in base of the sCTLA-4 values (low 0.1–10 ng/ml, and high >10.1 ng/ml). MIT3 was the only marker of PBC showing a significant positive correlation with the amount of sera sCTLA-4, both comparing low and high sCTLA-4 levels. In addition, a difference between AMA-positive and AMA-negative with low sCTLA-4 levels for PBC screen and sp100 was apparent. Dotted lines indicate the cut off value (25 Units), respectively for PBC screen, MIT3, gp210, and sp100.

In addition, we divided AMA-positive and AMA-negative PBC patients in two subgroups based on low (i.e. 0.1–10 ng/ml), and high sCTLA-4 concentration (i.e. >10.1 ng/ml) (Fig. 2). Of interest, MIT3 was the only marker showing a significant positive correlation with sCTLA-4, both comparing low and high sCTLA-4 levels (p<0.0001). A significant difference between AMA-positive and AMA-negative with low sCTLA-4 levels for PBC screen and sp100 was apparent (p<0.0001 and p = 0.0174, respectively). All the other clinical/functional parameters analysed failed to show any significant correlation with serum sCTLA-4 levels.

Of interest, when we stratified all PBC patients as low (i.e. 0.1–10 ng/ml), and high sCTLA-4 concentration (i.e. >10.1 ng/ml), we could observe a positive correlation between sCTLA-4 and the PBC screen test, IIF-AMA results, MIT3, and gp210 presence (Table 2). Otherwise, all the other clinical/functional parameters analysed failed to show any significant correlation with serum sCTLA-4 levels.

**Table 2:** Biochemical, serological and histological parameters from PBC patients (values are expressed as median and range) according to sCTLA-4 levels (low 0.1–10 ng/ml, and high >10.1 ng/ml).

	sCTLA-4 low	sCTLA-4 high	P value
	(n = 28)	(n = 55)	
Women (*n*)	24	50	n.s.
Age (*years*)	37–82	38–82	n.s.
PBC screen (*units*)	69 (2–178)	144.5 (3–200)	p<0.0001
AMA+ (*n*)	11	52	p<0.0001
MIT3 (*units*)	5 (1–139)	118.5 (1–300)	p<0.0001
gp210 (*units*)	2 (1–33)	4 (1–16)	p = 0.0007
sp100 (*units*)	4 (1–159)	4 (1–179)	n.s.
ALP (*UI/l*)	128 (55–2943)	339.5 (144–2675)	p = 0.0011
gammaGT (*U/l*)	67 (39–1822)	166.5 (37–1335)	n.s.
ALT (*UI/l*)	42 (32–555)	51 (37–159)	n.s.
Total bilirubin (*mg/dl*)	0.63 (0.3–11.7)	0.8 (0.5–41.4)	n.s.
Direct bilirubin (*mg/dl*)	0.15 (0.1–4.7)	0.2 (0.3–1.8)	n.s.
Albumin (*g/l*)	4.1 (2.9–5.3)	4.3 (3–5.8)	n.s.
Histological Scheuer stage	II (I–IV)	III (I–IV)	n.s.
I	8 patients	13 patients	
II	12 patients	13 patients	
III	7 patients	17 patients	
IV	2 patients	12 patients	

## Discussion

Increased serum levels of sCTLA-4 have been demonstrated in several autoimmune diseases [31]. The results of the present study showed for the first time that high levels of the soluble form of CTLA-4 are detectable in sera from patients with PBC displaying positivity for AMA. This did not occur in control subjects with viral (HBV) hepatitis, nor in normal individuals. The presence of sCTLA-4 was confirmed by Western blot, which allowed the demonstration of the caracteristic 23-kDa band in ELISA positive sera only. Although it was not possible to performe absortion experiments with specific monoclonal antibodies, we think this finding strongly support the specificity of our data.

Preliminary experiments (comparing 44 AMA-positive vs. 16 AMA-negative patients) demonstrated the presence of sCTLA-4 only in the sera of the first group of patients (data not shown) [32]. In order to better evidentiate this difference, we increased the number of AMA-negative patients included in this study: only patents who were negative for MIT3 the most relevant fine specificity responsible for AMA IIF reactivity, were considered. Interestingly, in this selected group of AMA-negative patients with histologically proven PBC, serum sCTLA-4 was almost invariably undetectable, as in control subjects. At variance, no relationship was found between sCTLA-4 concentration and positivity for other PBC-related autoantibodies directed against nuclear antigens, such as sp100 and gp210.

Altogether, these findings may prompt two types of considerations.

First of all, they are reminiscent of similar observations reported by a number of research groups, including our own, in other autoimmune diseases (both systemic and organ-specific) [26]–[31], [33], [34], and in other immunological or haematological disorders, such as IgE-mediated reaction to hymenoptera venom [35], or acute lymphoblastic leukemia (ALL) in paediatric patients [36]. In some of these conditions, sCTLA-4 concentrations appeared to be somehow correlated with disease activity or outcome (e.g. severity of histological lesion, and gluten exposure in patients with celiac disease) [34]. In addition, in a different model disease, a correlation was observed among CD1d expression and higher levels of sCTLA-4 in B-ALL patients, suggesting a possible role of this soluble molecule as a marker of progression to malignancy, or as a marker of severity of this neoplastic disease [36]. Moreover, *in vitro* experiments proved that the soluble form of CTLA-4 present in autoimmune sera is functionally active, namely, it is able to bind its physiological ligands and to interfere with cell-to-cell interaction crucial for the costimulation process (which in turn is crucial for mounting an efficient, as well as pathological immune response) [27, 31, 32 34–36]. The nature of such interference seems to depend upon the activation state of T-cells. Specifically, sCTLA-4 *in vitro* appeared to favor the expansion and cytokine production of chronically activated T-cells by blocking the triggering and negative signaling of their membrane CTLA-4, whereas sCTLA-4 interaction with naïve, membrane CTLA-4-negative T-cells had the opposite effect [27], [31], [32], [34]–[37]. Thus, the presence of sCTLA-4 was suggested to be a relevant mechanism of perpetuation of immunological injury, possibly somehow related to disease outcome. That this is not simply a nonspecific inflammatory phenomenon is suggested by the lack of sCTLA-4 rise in controls with infectious diseases. Of note, the possible role of sCTLA-4 in modulating tissue damage was also suggested in a murine model [38].

The second relevant consideration rised by the findings of the present study is the apparent relationship of sCTLA-4 with AMA reactivity. As AMA-negative PBC patients are rare, we selected a consistent group of such patients and compared their sCTLA-4 levels with a comparable AMA-positive group of patients. It is debated whether AMA-negative PBC patients represent or not a distinct subset of PBC: they account for 2 to 5% of total PBC patients [19] and it has been suggested that the relatively low sensitivity of IIF method for AMA is at least in part responsible for AMA negativity. Nevertheless, the limits of sensitivity of IIF explain only in part AMA-negative PBC. In fact, in our series we selected a relatively large group of AMA-negative/MIT3-negative patients. In addition, the comparison of functional data and histology seems to suggest that AMA-negative patients have a milder disease than AMA-positive ones. In fact, the distribution of patients according to the Scheuer stage shows a higher damage in AMA-positive (with the majority of III and IV stage) versus the AMA-negative ones (with the majority of I and II stage). The striking difference in sCTLA-4 serum concentrations in relationship to AMA presence does support the concept that a major immunological difference exist between the two groups of patients. In fact, some literature data underline the possible pathogenetic role of anti-mitochondrial reactivity *per se* via molecular mimicry [11]–[13], [17]–[19], and a meta-analysis on the role of *CTLA-4* SNP (+49A/G) as a predisposing factor in PBC suggests, though circumstantially, a link with AMA positivity [34].

However, several points have to be clarified. There is some doubt about the molecular heterogeneity of sCTLA-4 and the relationship between the ability to produce sCTLA-4 and the *CTLA-4* polymorphisms observed in some autoimmune diseases. In particular, no correlation has been recently found between three recurrent single nucleotide mutations, CT60 (rs3087243), +49 A/G (rs231775) and −318 (rs5742909) and sCTLA-4 production [38]. As mentioned above, a role of other related genes is likely, although no data are available to date. Finally, there is still a debate about the spliced/shaded nature of the soluble variant of CTLA-4 [27], [34], [36], [39]. Moreover, the relationship between *CTLA-4* polymorphism(s) described associated with autoimmune diseases (including PBC) [33] and the ability to produce the spliced soluble form of the molecule has been not yet elucidated.

## Conclusion

In conclusion, we have shown that serum sCTLA-4 values were elevated only in AMA (MIT3)-positive and not in AMA (MIT3)-negative PBC patients. Thus, some immunological difference of AMA-positive patients with respect to AMA-negative ones seems to exist. Data on sCTLA-4 in asymptomatic AMA-positive individuals could help to elucidate its possible role in the natural history of the disease.
